# The Impact of Old Age Pension Eligibility on Alcohol Consumption: Evidence From a Population-Based Study in Rural South Africa

**DOI:** 10.1093/geroni/igad136

**Published:** 2024-02-01

**Authors:** Janet Jock, Erika T Beidelman, Lindsay C Kobayashi, Stephen Tollman, Meredith Phillips, Chodziwadziwa Whiteson Kabudula, Molly Rosenberg

**Affiliations:** O’Neill School of Public and Environmental Affairs, Indiana University, Bloomington, Indiana, USA; Department of Epidemiology and Biostatistics, Indiana University School of Public Health, Bloomington, Indiana, USA; Department of Epidemiology, University of Michigan School of Public Health, Ann Arbor, Michigan, USA; MRC/Wits Rural Public Health and Health Transitions Research Unit (Agincourt), Faculty of Health Sciences, School of Public Health, University of the Witwatersrand, Johannesburg, South Africa; MRC/Wits Rural Public Health and Health Transitions Research Unit (Agincourt), Faculty of Health Sciences, School of Public Health, University of the Witwatersrand, Johannesburg, South Africa; Department of Epidemiology and Biostatistics, Indiana University School of Public Health, Bloomington, Indiana, USA; MRC/Wits Rural Public Health and Health Transitions Research Unit (Agincourt), Faculty of Health Sciences, School of Public Health, University of the Witwatersrand, Johannesburg, South Africa; Department of Epidemiology and Biostatistics, Indiana University School of Public Health, Bloomington, Indiana, USA; MRC/Wits Rural Public Health and Health Transitions Research Unit (Agincourt), Faculty of Health Sciences, School of Public Health, University of the Witwatersrand, Johannesburg, South Africa

**Keywords:** Alcohol, Health, Pension, South Africa, Sub-Saharan Africa

## Abstract

**Background and Objectives:**

Alcohol causes more than 3 million deaths a year globally and contributes to over 5% of global disease and injury. Heavy drinking and alcohol use disorders among older adults have increased in the last 10–15 years. For individuals living in low-income countries, where wages are low and unemployment is high, old age pensions may provide a significant increase in household income. In turn, the receipt of supplementary income may increase spending on alcohol. Earlier life factors and socioeconomic status may affect alcohol consumption, making it difficult to directly assess the impact of income on alcohol consumption. This study reduces the potential for endogeneity with other life factors by exploiting an exogenous increase in income from old age pensions to isolate the impact of extra income on alcohol consumption for older adults.

**Research Design and Methods:**

We used a regression discontinuity design to assess changes in drinking patterns among rural, low-income adults who were 3 years below and 3 years above South Africa’s Old Age Pension Grant eligibility threshold (age 60). We assessed this relationship separately by gender and for employed and unemployed individuals.

**Results:**

We observed a significantly increased alcohol use associated with the Old Age Pension Grant eligibility for employed men (β = 4.57, 95% confidence interval: 1.72–12.14). We did not observe this same trend for unemployed men or for women.

**Discussion and Implications:**

The analysis in this study indicates that increased income from reaching the pension eligibility age may contribute to an increase in alcohol consumption for employed men. Interventions, such as informational campaigns on the risks of alcohol consumption for older adults or age-appropriate health interventions to help individuals reduce alcohol consumption, targeted around the time of pension eligibility age for employed men may help to reduce alcohol-related harms in low-income, rural sub-Saharan African settings.

**Translational Significance**: Alcohol use disorders have increased in the last 10–15 years. For low-income individuals, pensions often provide a significant increase in income that may be associated with higher alcohol spending. Using a regression discontinuity design, this study observed significantly higher reported alcohol consumption for employed men after reaching old age pension eligibility age when compared to men before reaching old age pension eligibility age. These results underscore the importance of alcohol-related interventions, such as informational campaigns on the risks of alcohol consumption for older adults or health interventions to help individuals reduce alcohol consumption, around pension eligibility age for employed men.

## Background and Objectives

Alcohol causes more than 3 million deaths a year globally and contributes to over 5% of global disease and injury (World Health Organization, 2018). In addition to its direct adverse health impacts, such as cirrhosis and addiction, alcohol is a risk factor for several noncommunicable diseases such as diabetes, cancers, and cardiovascular disease (Rehm et al., 2017). Research on the “alcohol harm paradox” suggests that people in low socioeconomic status (SES) subpopulations seem to experience more harm from alcohol, even when they consume the same amount or less alcohol than higher SES groups (Probst et al., 2020). For example, Probst et al. (2020) find that individuals with low SES are 1.5–2 times more likely to die from alcohol-related causes than all causes, compared to those with high SES (Probst et al., 2020). The harms from alcohol use among low SES subpopulations may be exacerbated in low- and middle-income countries (LMICs) that lack sufficient health facilities to accommodate the heavy burden of alcohol-related diseases and associated adverse health consequences. Moreover, individuals are living longer, and, in turn, the older population is growing. Therefore, identifying the mechanisms through which alcohol consumption may be increasing for older adults in resource-limited settings can help us to identify potential national-level policy interventions to reduce alcohol intake among this population.

One possible reason for the current rise in alcohol-related mortality is the increase in alcohol use disorders among older adults in the last 10–15 years (Grucza et al., 2018; Han et al., 2017). This rise is detrimental for older adults due to their higher risks for chronic health conditions, medication use, and physical limitations that can be exacerbated by alcohol use. Alcohol consumption among older adults has been associated with an increased risk of adverse drug reactions (Onder et al., 2002), increased risk of social isolation and depressive symptoms (Choi & DiNitto, 2011), cognitive impairment and decline (Heymann et al., 2016; Hudetz et al., 2007; Thomas & Rockwood, 2001), and short-term mortality (Thomas & Rockwood, 2001).

Retirement is a transition that induces lifestyle changes (Wang et al., 2014). One of these changes may be in the quantity or frequency of alcohol consumption. However, much of the research on this relationship has produced mixed results (Ding et al., 2016; Oshio & Kan, 2017; Xue et al., 2020; Zantinge et al., 2014). Some studies have found that retirement is associated with an increase in alcohol use and heavy drinking (Henkens et al., 2008; Zins et al., 2011), whereas others have found reductions in alcohol consumption after retirement (Midanik et al., 1995; Rodriguez & Chandra, 2006). Because studies to date have produced mixed results on the impacts of retirement on alcohol consumption, it is necessary to understand the mechanisms underlying the association between alcohol consumption and reaching retirement age eligibility. This study is a valuable contribution to our understanding of these underlying mechanisms by examining how supplementary income from reaching old age pension eligibility may be associated with changes in alcohol consumption.

One potential mechanism through which retirement may affect alcohol use is the increased disposable income from old age pensions. The percent of preretirement income that a pension benefit replaces is referred to as the replacement rate (Steuerle et al., 2000). To maintain a similar standard of living, the International Labour Organization recommends a target replacement rate for pensions of 40% of the worker’s prior annual employment wages (International Labour Organization, 2022). This level is not typically reached in high-income countries. However, the replacement rate for US households on the lower quarter of the income distribution is typically equal to, or more than, their previous income (J. P. Smith, 2003). Thus, although old age pensions for high-income earners do not typically increase income postretirement, old age pensions for low-income earners may increase household income postretirement. For individuals living in countries where wages are low and unemployment is common, old age pensions may provide a significant increase in household income post-old age pension eligibility. This highlights the important redistributive impacts of old age pensions, particularly in low-income settings. Moreover, the South African old age pension is about 23% of gross domestic product, making it one of the largest pension programs in sub-Saharan Africa (World Bank, 2015). It is possible that this income increase provides more discretionary income to individuals that they may spend on alcohol.

### South Africa’s Older Persons Grant

The South African Older Persons Grant (also called the Old Age Pension Grant) is the country’s largest social assistance program. It was established in 1928 and expanded in the 1990s to include the country’s Black population. The Older Persons Grant is a means-tested program that does not require contribution or prior work participation. It provides a monthly cash transfer of R1980 ($112 USD) to eligible adults aged 60 years and older (International Labour Office, 2016). The pension amount is nearly twice the median per capita income for Black South Africans and provides access to credit markets for many households (Ralston et al., 2016). At the time of data collection for this study, individuals qualified for the program if their income was <R61,800 ($5,340) a year and had assets worth <R891,000 ($77,000; Ralston et al., 2016). This study uses reaching Old Age Pension Grant age eligibility at age 60 as a proxy for pension receipt. Because the sociodemographic characteristics of individuals in this setting are in line with the target requirements for the Old Age Pension Grant, it is likely that everyone above 60 years old in the sample was eligible with respect to the means testing.

Our study investigates the relationship between old age pension age eligibility and alcohol consumption for individuals living in a rural, low-income region of South Africa. Findings provide evidence for the importance of alcohol-related interventions around the time of reaching old age pension eligibility age for employed men.

## Method

### Study Population and Data Sources

This study analyzed data from the baseline wave of the population-based “Health and Ageing in Africa: A Longitudinal Study of an INDEPTH Community in South Africa” (HAALSI) cohort of 5,059 adults aged ≥40 years in 2014/2015 (Gómez-Olivé et al., 2018). HAALSI is a longitudinal study embedded within the Agincourt Health and Sociodemographic Surveillance Site (HDSS), an annual census covering nearly 100% of the underlying Agincourt research site (Kahn, Collinson, Gómez-Olivé, et al., 2012). HAALSI is designed to monitor risks for health conditions related to aging for these older residents of the Agincourt research site (Gómez-Olivé et al., 2018). Data from the HAALSI baseline wave used for this analysis were collected from 2014 to 2015 and consisted of 2,345 men and 2,714 women. Ethical approval for HAALSI was obtained from the University of the Witwatersrand Human Research Ethics Committee (#M141159), the Harvard T.H. Chan School of Public Health Office of Human Research Administration (#13-1608), and the Mpumalanga Provincial Research and Ethics Committee.

To exclude individuals who were far from pension eligibility age and increase the odds of exchangeability across exposed and unexposed groups, we created two subsamples for analysis. The first restricted the sample to individuals 3 years above and below the Old Age Pension Grant eligibility age threshold (ages 57–62; *N* = 455 for men and *N* = 502 for women), and the second restricted the sample to individuals 5 years above and below the Old Age Pension Grant eligibility age threshold (ages 55–64: *N* = 729 for men, *N* = 781 for women).

### Key Measures

Our exposure of interest was Old Age Pension Grant eligibility age, coded as a binary threshold indicator representing whether each study participant was below or above the age of Old Age Pension Grant eligibility at the time of the HAALSI baseline wave data collection. Individuals who were below 60 years old were assigned 0 whereas individuals who were ≥60 years old at the time of their HAALSI interview were assigned 1. As data on direct Old Age Pension Grant receipt are not available and HAALSI self-report data are inconsistent, we used Old Age Pension Grant age eligibility as a proxy for direct Old Age Pension Grant receipt. This is a plausible assumption as turning age 60 is a strict requirement to receive the Old Age Pension Grant. Additionally, due to the sociodemographic characteristics of the study population, participants in this study are highly likely to meet the means-tested income eligibility criteria. This is due to the high prevalence of poverty, unemployment, and food insecurity in this region (Kahn, Collinson, Gómez-Olivé, et al., 2012). Data for this study were collected from the Bushbuckridge subdistrict of the Mpumalanga Province of South Africa. At the time of data collection, 42.6% of individuals in the Mpumalanga province were below the poverty line (647 Rand or $51.45 USD per person per month; Kahn, Collinson, Xavier Gómez-Olivé, et al., 2012). This allows us to make an inference about the impacts of extra income from pension receipt by using pension eligibility age.

Our outcome of interest was the frequency of alcohol use, measured as the self-reported average number of alcoholic drinks consumed per month. Participants who reported that they currently drink alcohol were asked how often they consume at least one alcoholic drink. The HAALSI survey accounted for the varying levels of alcohol content in different drinks by clarifying for the participants what is considered “one drink.” The interviewer explained to study participants that “one drink” is considered equal to one shot of a strong alcoholic drink like spirits, or one full glass of a light alcoholic drink like beer. They were then asked how many days per week they drink and how many alcoholic drinks they consume on the days they drink. The response ranged from 0 to 150 alcoholic drinks per month. Individuals who reported drinking no alcohol were coded as consuming zero drinks per month. Responses were then coded as a continuous variable that estimated the average number of alcoholic drinks consumed per month.

We also included covariates in our analysis to control for sociodemographic differences across individuals above and below the Old Age Pension Grant eligibility age threshold: marital status (never married, divorced/separated, currently married, vs widowed), highest education level (none, some primary, some secondary, vs secondary or more), country of origin (South Africa or other), number of children, current employment (yes vs no), and wealth asset index. The wealth asset index variable used in this study was created from a principal components analysis of household characteristics and the ownership of household items (Gómez-Olivé et al., 2018). Proxying wealth by means of an asset index in low-income settings has been proven to be robust, with results approximating data on per capita poverty and output (Filmer & Pritchett, 2001). We included current employment in the covariate set, despite our Old Age Pension Grant eligibility age threshold, as there was significant variation in employment status across the levels of exposure. Reaching the age of pension eligibility was not directly correlated with current employment as some individuals continued to work past age 60. These individuals would still be eligible for the Old Age Pension Grant.

### Statistical Analysis

We adopted a Regression Discontinuity Design (RDD) to assess the association between reaching Old Age Pension Grant eligibility age and subsequent reported drinking frequency. The RDD is a quasi-experimental design where individuals are assigned to an intervention based on whether they are above or below a cutoff on a continuously measured, exogenous treatment assignment variable (Smith et al., 2017). The RDD sets up observational data in ways that approximate trial control in experimental groups. The RDD in this study compares individuals immediately above and below the Old Age Pension Grant eligibility age threshold based on their birthdate. Because birthdate in a small window is not plausibly related to other factors that may affect alcohol consumption, such as socioeconomic status, employment, or marital status, the RDD allows us to treat Old Age Pension Grant eligibility age as an exogenous treatment where individuals directly above and below the threshold cutoff likely do not differ significantly across key sociodemographic characteristics aside from date of birth. The binary Old Age Pension Grant eligibility age variable served as the discontinuity threshold in the RDD. We treated this as a sharp assignment of the discontinuity as individuals below the age of 60 are strictly not eligible to receive the Old Age Pension Grant. We should note that there is no “first stage” in our RDD approach, as is usually seen in RDD design analyses. Turning 60 results in sharp assignment of Old Age Pension Grant eligibility, because individuals are strictly eligible for the Old Age Pension Grant at this age. Hence, our RDD shows the effect of turning 60 years old and, in turn, becoming eligible to receive the Old Age Pension Grant. This makes it such that the first stage is one. This approach is similar to other cases of sharp RDDs (Hahn et al., 2001). Because the individuals in this study cannot manipulate the value of their age (and, in turn, Old Age Pension Grant eligibility), the outcome probability values at the cutoff point are as good as random.

Our analysis shows the discontinuity in the outcome probability (alcohol consumption) at the forcing variable (age). We stratified the sample by gender because South African men demonstrate much higher alcohol consumption than women (Andersson et al., 2018; Simbayi et al., 2004). In turn, we anticipated finding gender-differentiated impacts. We also stratified the analysis by employment status because some individuals continued to work past age 60 although they were still eligible for the Old Age Pension Grant. Moreover, we performed analyses across two age-restricted samples: (1) ±3 years from the point of discontinuity at 60 years (*n* = 455 for men and n = 502 for women) and (2) ±5 years from the point of discontinuity at 60 years (*n* = 729 for men, *n* = 781 for women). Restricting the sample in this way allowed us to maintain the sample size while preserving exchangeability. To maximize statistical power, we used the binary threshold indicator as the only exposure. Thus, we compared the group of individuals aged lower than the Old Age Pension Grant eligibility cutoff to the group of individuals aged above the Old Age Pension Grant eligibility cutoff for both restricted samples. This restricted model approximates a sample of individuals that only by chance fall on either side of the discontinuity and approximates a randomly assigned treatment with exchangeability across the comparison groups (Calonico et al., 2014; Smith et al., 2017). Based on analysis of covariates, the exchangeability assumption was met for the ±3-year sample, thus, we presented unadjusted models for this analysis. As a sensitivity analysis, and since the assumption of exchangeability was not met for the ±5-year sample, we presented adjusted models for this analysis. The purpose of this sensitivity analysis is to ensure that our results did not change drastically when adjusting for potentially influential demographic characteristics. Adjustment for these critical confounders is considered a doubly robust method and helps to further reduce bias in the results, especially considering that complete certainty around exchangeability assumptions is not possible.

To estimate the association between pension eligibility and alcohol consumption, we fit unadjusted and adjusted zero-inflated negative binomial regression models, stratified by gender and employment status. We used this model because the outcome variable, self-reported alcohol consumption, contained many zero values. Results of the models are presented as rate ratios representing the percent change in the average number of alcoholic drinks consumed per month associated with the exposure. The model for the ±5-year sample was adjusted for marital status, education level, wealth asset index, number of children, country of origin, and current employment status.

A key RDD assumption is that the probability of the outcome is continuous in the absence of the exposure (Smith et al., 2017). Therefore, trends in alcohol consumption before and after the Old Age Pension Grant eligibility age 60 cutoff point should be continuous. If our RDD satisfies this assumption, the only discontinuity in alcohol consumption should be at the age 60 cutoff point. To verify this assumption, we performed a robustness check, examining the average number of predicted drinks per month grouped by 5-year age intervals and stratified by employment status.

## Results

**Figure 1.**
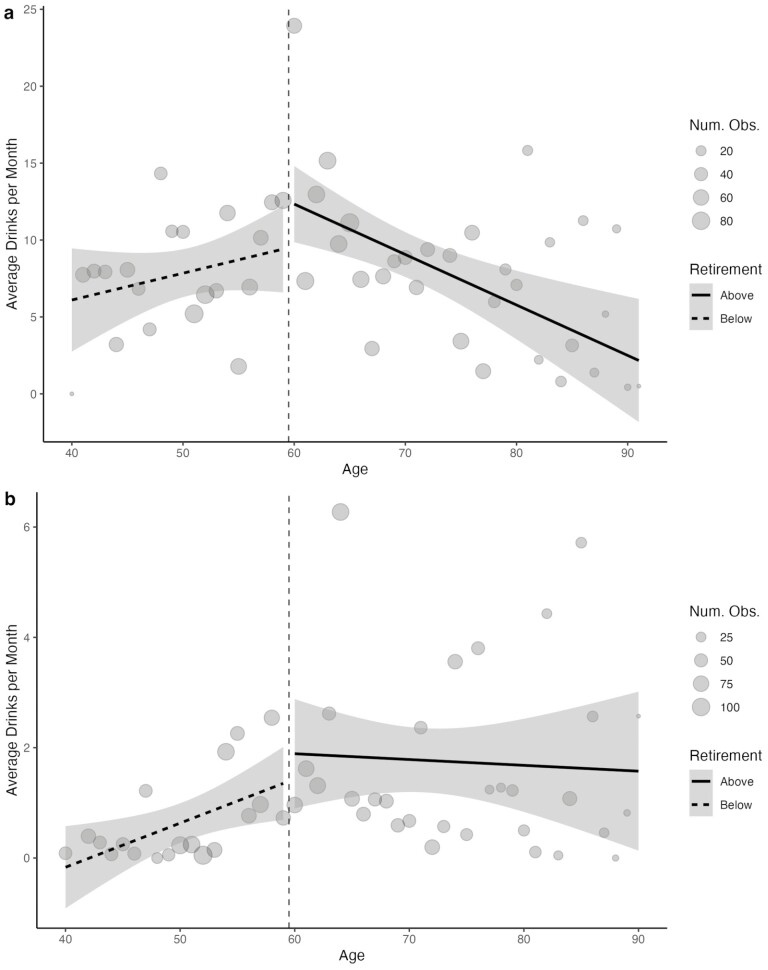
Plot of self-reported number of alcohol drinks per month across men (A) and women (B), below and above Old Age Pension Grant eligibility age. The *y*-axis scale for the plotted average number of drinks by age for men (A, top) has a much wider range (0–25) than the plotted average number of drinks by age for women (B, bottom; 0–6). This is because women, on average, reported much lower alcohol consumption than men and, therefore, the trends were difficult to see using a wider range. Num. Obs. = Number Observed. *Lines represent average trend in number of drinks above (blue) and below (red) the Old Age Pension Grant age threshold. Dots represent the number of observations reporting each number of drinks per month at each age.

Table 1 shows the demographic characteristics of our restricted sample of 957 individuals between the ages of 57 and 62 years. Nearly four fifths of the individuals had a primary education or less and were unemployed. There were no meaningful differences across the comparison groups when restricting the age groups to 3 years above and below the Old Age Pension Grant eligibility age threshold. Supplementary Table S1 shows the demographic characteristics for individuals above and below the Old Age Pension Grant eligibility age threshold for the full sample. Although a discontinuity in the mean self-reported number of alcoholic drinks per month is observed across the Old Age Pension Grant eligibility age threshold for men, no such discontinuity is evident for women (see Figure 1). Figure 1A demonstrates an upward trend in alcohol intake for men with increasing age before the Old Age Pension Grant eligibility age threshold. However, the largest point of discontinuity in alcohol consumption can be observed between ages 59 and 60 (four more average drinks per month). After age 60, the mean number of self-reported drinks per month declines for men. Figure 1B demonstrates the self-reported number of alcoholic drinks per month for women below and above the Old Age Pension Grant eligibility age threshold. Unlike the trend we saw for men, there is almost no trend of increased alcohol consumption for women below or at the Old Age Pension Grant eligibility age threshold (<1 more average drinks per month). Moreover, after age 60, the mean number of self-reported drinks per month remains the same across age for women.

**Table 1.** Descriptive Statistics Across Men and Women Above and Below the Old Age Pension Grant Eligibility Age Threshold, Restricted Age Sample (Ages 57–62).

Characteristic	Overall	Pre-Old Age Pension Grant eligibility	Post-Old Age Pension Grant eligibility	*p* Value
	*n* = 957	*n* = 393	*n* = 393	
Asset index, mean (*SD*)	1.67 (2.5)	1.71 (2.6)	1.64 (2.4)	.6
Consumption index, mean (*SD*)	3.05 (1.5)	2.86 (1.5)	3.20 (1.4)	.02
Male, *n* (%)	455 (48%)	175 (45%)	280 (50%)	.1
Number of children, mean (*SD*)	5.23 (2.7)	5.28 (2.9)	5.19 (2.5)	.9
Missing, *n*	57	22	35	
Highest education, *n* (%)				.2
None	404 (42%)	164 (42%)	240 (43%)	
Some Primary/secondary	504 (53%)	204 (52%)	300 (53%)	
Secondary or more	45 (4.7%)	24 (6.1%)	21 (3.7%)	
Missing, *n*	4	1	3	
Country of origin, *n* (%)				.03
South Africa	703 (74%)	274 (70%)	429 (76%)	
Other	253 (26%)	119 (30%)	134 (24%)	
Missing, *n*	1	0	1	
Current employment, *n* (%)				<.001
Employed	162 (17%)	90 (23%)	72 (13%)	
Unemployed	696 (73%)	261 (67%)	435 (77%)	
Missing	96 (10%)	41 (10%)	55 (9.8%)	
Marital status, *n* (%)				.7
Never married	41 (4.3%)	18 (4.6%)	23 (4.1%)	
Separated/divorced	146 (15%)	57 (15%)	89 (16%)	
Widowed	266 (28%)	104 (26%)	162 (29%)	
Currently married	503 (53%)	214 (54%)	289 (51%)	
Unknown, *n*	1	0	1	
Household size, *n* (%)				.7
Alone	113 (12%)	44 (11%)	69 (12%)	
2 persons	75 (7.8%)	35 (8.9%)	40 (7.1%)	
3–6 persons	428 (45%)	176 (45%)	252 (45%)	
7+ persons	341 (36%)	138 (35%)	203 (36%)	

*Note*: *SD* = standard deviation.

Within the sample ±5 years from the eligibility threshold, employed men who were above the Old Age Pension Grant eligibility age threshold reported drinking 4.95 times more alcoholic beverages per month than those below the Old Age Pension Grant eligibility age threshold (see Table 2, β = 4.95, 95% confidence interval [CI]: 2.32–10.56). There was no statistically significant change in alcohol consumption for unemployed men above and below the Old Age Pension Grant eligibility age threshold (see Table 2, β = 1.33, 95% CI: 0.71–2.50). Among women, there was no statistically significant change in alcohol consumption above and below the Old Age Pension Grant eligibility age threshold, even when stratifying across current employment status (see Table 2, employed women: β = 1.05, 95% CI: 0.26–4.23, unemployed women: β = 0.82, 95% CI: 0.15–4.41).

**Table 2.** Effect of Old Age Pension Grant Eligibility Age on Average Number of Monthly Alcoholic Drinks for Men and Women and Stratified By Employment Status, ±5 Years From Eligibility Threshold

Employment status	Men			Women		
	Estimates	CI	*p*	Estimate	CI	*p*
All^a^	**1.96**	**1.15–3.34**	.01	0.46	0.09–2.38	.4
Employed^b^	**4.95**	**2.32–10.56**	**<.001**	1.05	0.26–4.23	.9
Unemployed^b^	1.33	0.71-2.50	.4	0.82	0.15–4.41	.8

*Notes:* Model for employed women was only adjusted for asset index and number of children due to low sample size *(n* = 75) and nonpositivity. Values in bold indicate statistical significance. CI = confidence interval.

^a^Adjusted for current working status, asset index, education level, marital status, country of birth, and number of children.

^b^Adjusted for asset index, education level, marital status, country of birth, and number of children.

Within the sample ±3 years from the eligibility threshold, employed men who were above the Old Age Pension Grant age eligibility threshold reported drinking 4.57 times more alcoholic beverages per month than those below the Old Age Pension Grant eligibility age threshold (see Table 3, β = 4.57, 95% CI: 1.72–12.14). There was no statistically significant change in alcohol consumption for unemployed men above and below the Old Age Pension Grant eligibility age threshold (see Table 3, β = 0.75, 95% CI: 0.38–1.48). Moreover, there was no statistically significant change in alcohol consumption above and below the Old Age Pension Grant eligibility age threshold for employed or unemployed women (see Table 3, employed women: β = 1.00, 95% CI: 0.40–2.52, unemployed women: β = 1.35, 95% CI: 0.34–5.32). Although our results show a slightly stronger significance for the ±5-year sample compared to the ±3-year sample, the magnitude of effect is reasonably similar in both analyses with overlapping CIs. The limited sample size in the ±3-year analysis reduces the precision of the estimate for this subgroup.

**Table 3.** Effect of Old Age Pension Grant Eligibility Age on Average Number of Monthly Alcoholic Drinks for Men and Women and Stratified by Employment Status, ±3 Years From Eligibility Threshold

Employment status	Men			Women		
	Estimates	CI	*p*	Estimates	CI	*p*
All	1.19	0.68–2.09	.5	1.32	0.37–4.67	.7
Employed	**4.57**	**1.72–12.14**	**.02**	1.00	0.40–2.52	.9
Unemployed	0.75	0.38–1.48	.4	1.35	0.34–5.32	.7

*Notes:* Models are unadjusted due to assumed exchangeability between groups above and below the Old Age Pension Grant eligibility age threshold due to the ±3-year age restriction. Values in bold indicate statistical significance. CI = confidence interval.

**Figure 2.**
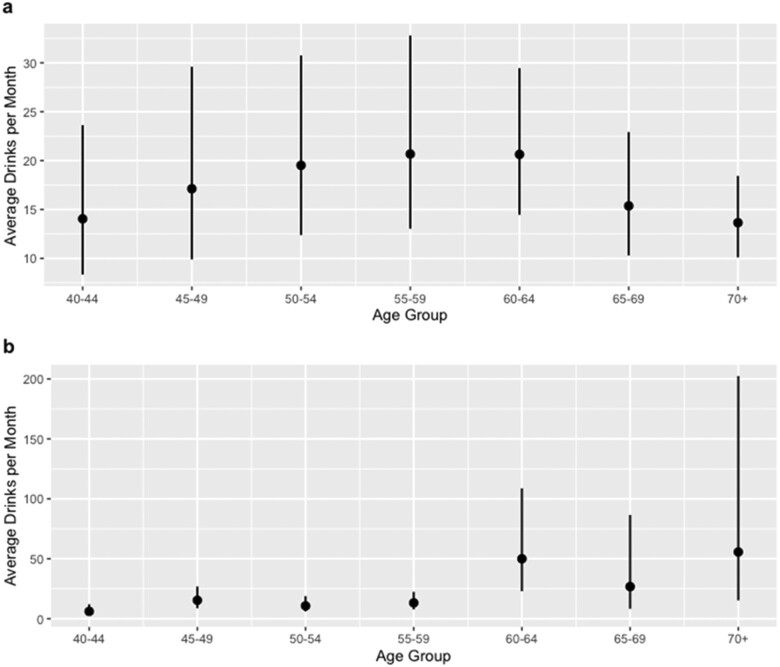
Robustness check comparing predicted number of drinks per month across 5-year age categories for unemployed (A) and employed (B) men.

For the robustness check, Figure 2A shows a slight increase in alcohol consumption for unemployed men from age 40 to 55. However, no change in alcohol consumption was observed for unemployed men between the ages of 55 and 64. In contrast, Figure 2B shows no change in alcohol consumption for employed men between the ages of 40 and 59. However, we see a discontinuity in alcohol consumption between the age groups 55–59 and 60–64, with the men who were employed after the Old Age Pension Grant eligibility age threshold consuming more than 25 drinks per month than the employed men below the Old Age Pension Grant eligibility age threshold. After age 64, this trend decreases. This robustness check provides evidence that the Old Age Pension Grant eligibility age threshold is the primary point of discontinuity in drinking levels for employed men in the HAALSI cohort. Moreover, alcohol consumption for employed men decreases by nearly 25 drinks in the 65–69 years age category. This trend may provide evidence of an income effect on alcohol consumption for employed men after reaching Old Age Pension Grant eligibility age in the short term that then tapers off in the long term as they adjust to the new income increase and possibly use the extra income to buy things other than alcohol.

## Discussion and Implications

This study assessed differences in alcohol drinking patterns in the 3 and 5 years before and after reaching Old Age Pension Grant eligibility age in a population-based study of older men and women in rural South Africa. When we stratified the sample by employment status, we found a positive association between reaching the Old Age Pension Grant eligibility age threshold and alcohol consumption for employed men. Employed men at and up to 3 years above the Old Age Pension Grant age eligibility threshold of 60 years reported consuming nearly five times more alcoholic beverages per month than employed men below the threshold. We did not observe changes in alcohol consumption among unemployed men or among women after reaching the Old Age Pension Grant eligibility age threshold. These results demonstrate evidence that the extra income from old age pension may have influenced an increase in alcohol consumption for employed men in this low-income, rural setting in sub-Saharan Africa.

Few studies have directly examined the impact of social assistance in the form of noncontributory cash transfers on the subsequent alcohol use of recipients and the existing evidence base is mixed. One randomized control trial (RCT) from the United States found that receipt of an unconditional cash transfer did not increase spending on alcohol, tobacco, or drugs among low-income mothers (Yoo et al., 2022). Other studies support the claim that cash transfers do not increase spending on tempting goods such as alcohol (Evans & Popova, 2014; Flaminiano, 2021; Handa et al., 2018; Marinescu, 2018). However, an analysis of cash assistance programs in Peru did find that the receipt of cash transfers was associated with small increases in spending on alcohol and sugar-sweetened products (White & Basu, 2016). Another study of universal cash transfer payments in the United States found an increase in nonmedical drug substance abuse the day after payment (Watson et al., 2020). Our results align with the US RCT because we found no association between pension eligibility and alcohol consumption for women. Moreover, our findings align with those from Peru, a similar LMIC setting. It is important to note that neither of these studies focused on older adult populations. Our study provides new evidence on the role of pension eligibility on alcohol consumption among older men and women in rural, sub-Saharan Africa.

There are several reasons why we may see an increase in alcohol consumption for employed men when they reach Old Age Pension Grant eligibility age; however, we do not see an increase for unemployed men. Because unemployed men do not have another source of income with which to pay for household necessities, the extra income from the old age pension may be used to pay for household needs. Employed men, on the other hand, have a source of income other than the old age pension with which to pay for household necessities before and at the time they reach the Old Age Pension Grant eligibility age. In turn, these men may have more discretionary income at their disposal when they begin to receive the extra Old Age Pension Grant income. They may choose to spend this extra income on leisure items including alcohol. In contrast, we do not find evidence for increased alcohol consumption for employed or unemployed women when they reach the Old Age Pension Grant eligibility age. This is likely because women of all age groups in this study reported very low levels of alcohol consumption. This finding for women is supported by other studies in South Africa that demonstrate higher levels of alcohol consumption, in general, for men than for women (Andersson et al., 2018; Simbayi et al., 2004) and studies in other settings that find substantially less spending on alcohol and tobacco among women than among men (; Case & Deaton, 1998; Sullivan et al., 2010). Studies have also shown that women spend cash transfer payments differently than men and sometimes in ways that benefit other household members, especially children (Burns et al., 2005; Khoza et al., 2018).

Importantly, alcohol consumption in this study was self-reported. Self-reported data are prone to both recall bias and social desirability bias which could result in under- or overreporting of our outcome variable. Underreporting of alcohol consumption is a well-known phenomenon and is the most common type of inconsistency between self-reported and biomarker-based measures of alcohol consumption (Grüner Nielsen et al., 2021). The underreporting of alcohol consumption frequency has been shown to be higher for men than for women (Sommers et al., 2000). Thus, due to this tendency to underreport, it is likely that the effect estimates for men’s alcohol consumption in our analysis were conservative and that the true effect estimates may be larger.

Our analysis was strengthened by using an RDD which helped to achieve exchangeability across the individuals before and after reaching Old Age Pension Grant eligibility age. Our results remained consistent when restricting the sample to an unadjusted 3-year age period on either side of the Old Age Pension Grant eligibility age threshold. Although we have reason to believe that most individuals were eligible to receive old age pension at age 60 due to the low SES study setting, this is an intent-to-treat analysis because we do not actually measure old age pension receipt. Moreover, we could not empirically test whether the outcome probability was continuous in the absence of the exposure. That said, we performed a robustness check comparing the predicted number of drinks per month across 5-year age categories for men. The robustness check demonstrated that there were not pre trends in alcohol consumption for any age group before the Old Age Pension Grant eligibility age threshold for employed men. The only discontinuity in alcohol consumption observed was below and above the Old Age Pension Grant eligibility age cutoff.

## Conclusion

Most of the current research on the effects of old age pension on alcohol use takes place in high-income countries. Our study is unique in that it focuses on old age pension eligibility for individuals living in a rural area in a low-income setting. In this population-based study of older adults in a rural, low-income region of South Africa, we observed a spike in alcohol consumption at the age of Old Age Pension Grant eligibility for employed men, but not for unemployed men. Moreover, we did not see changes in alcohol consumption across age groups for women, regardless of employment status. It is possible that this exogenous increase in income from the Old Age Pension Grant increased discretionary income for some employed men which, in turn, was spent on alcohol. As populations continue to grow and age in LMICs, and as the burden of alcohol-related diseases increases in older adults, it is crucial to identify the potential causes of increased alcohol consumption for older adults in resource-limited settings. These findings provide evidence around the importance of alcohol-related interventions at the time of reaching pension age eligibility for employed South African men. Promising interventions include informational campaigns on the risks of alcohol use such as SHARE (Senior Health and Alcohol Risk Education; Ettner et al., 2014), screening for alcohol use and physician feedback (Fink et al., 2005; Fleming et al., 1999), and age-appropriate psychological treatment interventions such as family interventions, motivational counseling, and cognitive–behavioral therapies (Sorocco & Ferrell, 2006). These age-appropriate interventions have demonstrated positive impacts in terms of reducing alcohol consumption among older adults.

## Supplementary Material

igad136_suppl_Supplementary_Table
