# Supplementary material for: The Impact of Old Age Pension Eligibility on Alcohol Consumption: Evidence From a Population-Based Study in Rural South Africa
Source: Innov Aging. 2024 Feb 1;8(4):igad136. doi: 10.1093/geroni/igad136 (PMC11020216; doi:10.1093/geroni/igad136)
Supplement: igad136_suppl_Supplementary_Table [file igad136_suppl_supplementary_table.docx]

**Online Supplementary Materials:**

**Table S1: Descriptive statistics across men and women above and below the Old Age Pension Grant eligibility age threshold**

| Characteristic | Overall | Pre-Old Age Pension Grant eligibility | Post-Old Age Pension Grant eligibility | P-value |
| --- | --- | --- | --- | --- |
|  | N = 1,511 | n = 638 | n = 873 |  |
| Asset Index, mean (SD) | 1.64 (2.5) | 1.76 (2.6) | 1.55 (2.4) | 0.2 |
| Consumption Index, mean (SD) | 2.99 (1.4) | 2.91 (1.5) | 3.06 (1.4) | 0.2 |
| Age (years), mean (SD) | 60.2 (3.2) | 57.0 (1.4) | 62.6 (1.7) | <0.001 |
| Male, n (%) | 729 (48%) | 297 (47%) | 432 (49%) | 0.3 |
| Number of Children, mean (SD) | 5.20 (2.6) | 5.10 (2.7) | 5.27 (2.5) | 0.1 |
| Missing, n | 93 | 38 | 55 |  |
| Highest Education, n (%) |  |  |  | <0.001 |
| None | 670 (45%) | 253 (40%) | 417 (48%) |  |
| Some Primary | 609 (40%) | 244 (38%) | 365 (42%) |  |
| Some Secondary | 148 (9.8%) | 86 (14%) | 62 (7.1%) |  |
| Secondary or More | 77 (5.1%) | 52 (8.2%) | 25 (2.9%) |  |
| Missing, n | 7 | 3 | 4 |  |
| Country of Origin, n (%) |  |  |  | 0.9 |
| South Africa | 1,088 (72%) | 458 (72%) | 630 (72%) |  |
| Other | 421 (28%) | 179 (28%) | 242 (28%) |  |
| Missing, n | 2 | 1 | 1 |  |
| Current Employment, n (%) |  |  |  | <0.001 |
| Employed | 386 (26%) | 199 (31%) | 187 (21%) |  |
| Unemployed | 1,120 (74%) | 437 (69%) | 683 (79%) |  |
| Missing | 5 | 2 | 3 |  |
| Marital Status, n (%) |  |  |  | 0.1 |
| Never Married | 62 (4.1%) | 30 (4.7%) | 32 (3.7%) |  |
| Separated/Divorced | 222 (15%) | 91 (14%) | 131 (15%) |  |
| Widowed | 411 (27%) | 155 (24%) | 256 (29%) |  |
| Currently Married | 814 (54%) | 361 (57%) | 453 (52%) |  |
| Unknown, n | 2 | 1 | 1 |  |
| Household Size, n (%) |  |  |  | 0.8 |
| Alone | 165 (11%) | 65 (10%) | 100 (11%) |  |
| 2-person | 126 (8.3%) | 51 (8.0%) | 75 (8.6%) |  |
| 3-6 person | 692 (46%) | 296 (46%) | 396 (45%) |  |
| 7+ person | 528 (35%) | 226 (35%) | 302 (35%) |  |
